# Bacteriophages Can Make a Difference in Water Quality: Evidence From a Community-Based Study From North India

**DOI:** 10.7759/cureus.27551

**Published:** 2022-08-01

**Authors:** Prerna Srivastava, C. P Mishra, Gopal Nath

**Affiliations:** 1 Community Medicine, Institute of Medical Sciences, Banaras Hindu University, Varanasi, IND; 2 Microbiology, Institute of Medical Sciences, Banaras Hindu University, Varanasi, IND

**Keywords:** mpn count, water quality, under-five children, rural area, diarrhoea, bacteriophage

## Abstract

Background and objectives

Diarrhoea is a preventable and treatable faecal-oral disease. Despite significant inputs from the health and non-health sector in the treatment and prevention of diarrhoea, it remains a significant contributor to under-five-years children mortality and exerts profound effects on their growth and development. Bacteriophage has the potential to prevent diarrhoea. Bacteriophage status may influence the extent of diarrhoea. The objectives of the study were a) to assess the bacteriophage status and quality of water based on the Most Probable Number (MPN) count in the drinking water of under-five years children, and b) to find the association of the extent of diarrhoea with the bacteriophage status and quality of drinking water of under-five-years children in rural areas of Varanasi.

Methods

This is a community-based cross-sectional study done in the Chiraigaon community development block in a rural area of Varanasi. Water samples were collected and analysed in the Department of Microbiology, Institute of Medical Sciences, Banaras Hindu University, Varanasi.

Results

The result of the study was that bacteriophage is present in 118 samples of water, whereas bacteriophage for *Escherichia coli *(*E. coli*),* Klebsiella,* and *Vibrio *were present in the drinking water of 81.1%, 53.8%, and 25.8% of under-five-years children, respectively. The water quality was highly satisfactory in 41.7% of samples and unsatisfactory in 15.2% of samples (p<0.004). All samples with highly satisfactory water quality had bacteriophages. Diarrhoea was present in 57.14% of samples without bacteriophage and 24.5% in samples with bacteriophage (p<0.01). The unadjusted odds ratio is 4.09.

Interpretation and conclusions

The odds of diarrhoea are four times higher in the water sample without bacteriophage than in the water sample in which bacteriophage is present. Bacteriophage study in preventing diarrhoea in children under five and health risk assessment call for focus.

## Introduction

The rising burden of non-communicable diseases has shifted the focus of health researchers from communicable diseases to non-communicable diseases. However, childhood diarrhoea remains a significant public health problem even now. The diarrhoeal disease control programme was launched by World Health Organization (WHO) in 1978 aiming to reduce mortality and malnutrition due to diarrhoea. In developing countries mortality due to diarrhoea has reduced significantly from nearly 5 million deaths per year in under-five-years children in 1983 to 446,000 deaths in 2016 [1,2]. Thus, diarrhoea still remains one of the major reasons of morbidity and malnutrition in under-five children. The strategy for diarrhoea control consists of case management, improved maternal and child health care, improved use and maintenance of drinking water and sanitation facilities and improved food hygiene, and detection and control of epidemics. It is estimated that in under-five children, 297,000 diarrhoea cases are attributable to unsafe water supply and sanitation, representing 5.3% of all deaths in this age group [3]. For infectious diarrhoea, the agents responsible are viruses, bacteria, protozoa, and helminths. Bacteria are responsible for about 45% of diarrhoea. In the ranking of the number of episodes of the cause of diarrhoea, the bacteria causing diarrhoea are *Escherichia coli *(*E. Coli*), *Campylobacter jejuni*, *Shigella*, *Salmonella *and *Vibrio *El Tor [4]. It is estimated that 63,000 annual deaths are due to *E. coli *infections; this is added to 5 million years of life lost (YLL) and 5 million disability-adjusted life years (DALY), which further compounds the suffering caused by this pathogenic contaminant [5].

Adequate drinking water and sanitation play a key role in preventing many more diseases, improving nutritional outcomes and for the provision of quality care in healthcare settings. The provision of safe and wholesome water has always been the target of the United Nations; the Sustainable Development Goal (SDG) 6 emphasises ensuring access to safe water and sanitation for all [6]. Worldwide, one in three persons does not have access to safe water for drinking and two out of five persons face challenges to find a safe facility for hand washing [7]. Safe and wholesome water should be assessably affordable and based on sustainable technology for preventing contamination and disinfection. Conventional disinfection and microbial control approaches are often insufficient to keep up with the increasing complexity and renewed relevance of this pressing challenge. Disinfectants such as chlorine cannot easily penetrate and eradicate biofilms and are also relatively ineffective against resistant microorganisms [8]. Phage treatments have the potential to control environmental wastewater process problems such as: foaming in activated sludge plants; sludge dewaterability and digestibility; pathogenic bacteria; and reduce competition between nuisance bacteria and functionally important microbial populations [9]. In addition, phages could also mitigate harmful cyanobacterial blooms that produce toxins in source waters, and could also serve as substitutes for the prophylactic use of antibiotics and biocides further preventing antibiotic resistance [8].

Bacteriophages are bacteria-killing viruses. The process of killing can be lytic and lysogenic. When phages multiply vegetatively they kill their hosts and the life cycle is referred to as the lytic life cycle. On the other hand, some phages known as temperate phages can grow vegetatively and can integrate their genome into the host chromosome replicating with the host for many generations [10].

With this background this study was conducted in a rural area of Varanasi, India, with the specific objectives of a) to assess the bacteriophage status and quality of water on the basis of the Most Probable Number (MPN) count in the drinking water of subjects, b) to examine linkages of the extent of diarrhoea with the quality and bacteriophage status of drinking water of the subjects.

## Materials and methods

This community-based cross-sectional study was carried out in the rural area of Varanasi, India. The primary concern of this study was the quality of drinking water. According to a pilot study in a non-study area, the water quality of 30% of under-five-years children was either suspicious or not satisfactory. Taking this as prevalence, the design effect of 1.5 and the dropout rate of 10%, the estimated sample size became 135. However, information was obtained for 132 under-five (0-59 months) children. The selection of subjects was done by adopting a multi-stage sampling procedure. In stage 1, the selection of the Chiraigaon community development (CD) block was done as the study block out of eight CD blocks of Varanasi by simple random sampling. In stage 2, all 134 villages of Chiraigaon were stratified into three strata according to their distance (viz. 5 km, 5 - 10 km, and >10 km) from the block headquarters. One village was selected from each stratum by stratified random sampling. In stage 3, the selected village's total enumeration of under-five-year children was done. This served as the sampling frame. The required number of under-five-year children taken as study subjects were selected from the three villages by adopting simple random sampling, according to probability proportional sampling to size.

The sources of drinking water of the study subjects were tap water, handpump, and in some cases, wells, in isolation as well as in combination. The mouth of the handpump was sterilized with a Bunsen burner or candle flame or match stick. First, the first 60 seconds of water flow was discarded; then the outflowing water was collected in sterilized wide-mouth bottles and capped. The bacteriophage status and the quality of the drinking water of the study subjects were assessed using scientifically valid tools and techniques [11]. The bacteriological quality of drinking water was assessed through the Most Probable Number (MPN) count for coliforms and the status of bacteriophage for *E. coli*, *Klebsiella *and *Vibrio*. The MPN count is calculated through presumptive coliform count (multiple test tube method). The water sampling procedure and method for bacteriological examination of water were done as per the standard protocol of WHO [12]. The quality of drinking water of the subjects was categorized with a McCrady chart as highly satisfactory (MPN<1), satisfactory (MPN 1-2 ), suspicious (MPN 3-10), and unsatisfactory (MPN >10). Information regarding diarrhoea experienced by the study subjects (under-five children) in the previous two weeks was obtained by interviewing the caregivers of the study subjects.

Ethical clearance

The study was done after taking ethical clearance from the Institutional Ethical Clearance (IEC) Board of the Institute of Medical Sciences, Banaras Hindu University (Dean/2018/EC/297) and consent was taken from the parents of the study subjects.

Statistical method

The data collected were entered into a personal computer and analysed using IBM SPSS software trial version 23 (IBM Corp, Armonk, USA). For inferential purposes, the Chi-square test was used. Association between two variables was considered as significant when the p-value was less than 0.05.

## Results

In this study, the bacteriophage status and drinking water quality of 132 under-five children were assessed following a standard technique. In these subjects, the extent of diarrhoea was also assessed in the previous two weeks (14 days). Bacteriophages were present in the drinking water of 118 out of 132 subjects.

Bacteriophages for *E. coli*, *Klebsiella, *and *Vibrio *were present in the drinking water of 81.1%, 53.8%, and 25.8% of subjects, respectively (Table 1).

**Table 1:** Bacteriophages status of drinking water of study subjects (N = 132)

Particular	Present		Absent		Total	
	No.	%	No.	%	No.	%
Bacteriophage for E.coli	107	81.1	25	18.9	132	100
Bacteriophage for Klebsiella	71	53.8	61	56.2	132	100
Bacteriophage for Vibrio	34	25.8	98	74.2	132	100

Out of 132 water samples, 41.7% were highly satisfactory (MPN<1) and 29.5% were satisfactory (MPN 1-2). In the case of 13.6% and 15.2% of water samples from the study subject, the quality of water was suspicious (MPN 3-10) and unsatisfactory (MPN >10 ), respectively (Table 2).

**Table 2:** The quality of water based on the Most Probable Number count

Class of Water	No.	%
Highly satisfactory	55	41.7
Satisfactory	39	29.5
Suspicious	18	13.6
Unsatisfactory	20	15.2
Total	132	100

Diarrhoea was present in 57.1 % of samples without bacteriophages and 24.5% in samples with bacteriophages. There existed a significant association (p<0.01) between bacteriophage status and the extent of diarrhoea (Table 3). The unadjusted odds ratio is 4.09 for subjects without the presence of bacteriophage in drinking water. 

**Table 3:** Association of diarrhoea with bacteriophage status

Status of Bacteriophage	Diarrhoea Present		Diarrhoea Absent		Total		Test of Significance
	Number	Percent	Number	Percent	Number	Percent	
Absent	8	57.2	6	42.8	14	100	x^2 ^ = 6.580 Df = 1 p= 0.010
Present	29	24.6	89	75.4	118	100	

Diarrhoea was found to be significantly (p<0.01) associated with the class of water. As the quality of water declined, the extent of diarrhoea increased significantly (p<0.01). As much as 12.7%, 30.7%, and 44.4% of subjects with water quality as highly satisfactory, satisfactory, and suspicious had diarrhoea, respectively. Half of the subjects with unsatisfactory water quality experienced diarrhoea in the last two weeks (Table 4).

**Table 4:** The extent of diarrhoea vis-à-vis the quality of drinking water of the study subjects

Class of water	Diarrhea Present		Diarrhea Absent		Total		Test of Significance
	Number (37)	Percent	Number	Percent	Number	Percent	x^2 ^= 13.42 Df = 3 P = 0.004
Highly satisfactory	7	12.7	48	87.3	55	100	
Satisfactory	12	30.7	27	69.3	39	100	
Suspicious	8	44.4	11	55.6	18	100	
Unsatisfactory	10	50.0	10	50	20	100	

The presence of bacteriophage in a sample of water used for drinking is significantly associated with the class of water as classified by the Most Probable Number (MPN). Bacteriophage was present in all samples of highly satisfactory followed by that of satisfactory and minimum in the not satisfactory class of water. The quality of drinking water is significantly associated with bacteriophage presence (Table 5).

**Table 5:** The association of bacteriophage status with the class of drinking water of the subjects

Class of water	Bacteriophage Present		Bacteriophage Absent		Test of Significance
	Number	Percent	Number	Percent	X^2^ =25.21 Df = 3 p = 0.00
Highly satisfactory	55	47.0	0	0	
Satisfactory	28	23.1	11	80	
Suspicious	18	15.4	0	0	
Not satisfactory	17	14.5	3	20	
Total	118	100	14	100	

## Discussion

Bacteriophages specifically target bacteria but not mammalian cells. They are strain-specific and phages are not self-propelled; they need to physically come in contact with bacteria to infect them. According to Ernest Hankin, the presence of mocked antibacterial activity against vibrio cholera in the waters of the Ganges and Yamuna rivers of India was due to an unidentified substance that passes through the porcelain filters and was heat labile [13]. About 20 years after Hankin, in 1915, Felix d’Herelle discovered bacteriophages. He used bacteriophages to halt outbreaks of cholera in India and plague in Egypt [14] The discovery of Penicillin in 1942 shifted the focus of research toward antibiotics [15]. The increase in the resistance of bacteria to antibiotics has led to an urgent need for a new area of therapeutic treatment and a new era of preventive treatment. Bacteriophages multiply at the site of infection and if bacteria become resistant to phages then phages do evolve naturally to infect the resistant bacteria, thus decreasing the chances of bacterial escape along with host-specificity, self-amplification, and low toxicity to humans [16].

In this study, the water quality of nearly four out of 10 subjects was highly satisfactory, whereas it was unsatisfactory in two out of 13 subjects. In the case of three out of 10 subjects, the water quality was either suspicious or unsatisfactory. Contrary to this study, higher unsatisfactory water quality was reported in some studies conducted in India [12,17,18].

In the present study, diarrhoea in under-five children is significantly associated with the class of water. It was maximum in subjects with the not satisfactory class of water (four out of 15) and minimum with the highly satisfactory class of water (nearly one out of 5). This is similar to a study conducted in Delhi, where six out of 10 children with diarrhoea were consuming unsatisfactory water, 27.7% of the children were drinking water that was of questionable quality, and only one out of 10 children were drinking water that was satisfactory and fit for human drinking purpose [12].

In this study, bacteriophages were isolated from water used for drinking, which is similar to a study conducted in Israel by Amron [19] and Amron and Kott [20], where bacteriophages were found for *E. coli *and *Bacteroides fragilis*. In another study, the presence of bacteriophages in drinking water points to inadequate water treatment or contamination at the point of use.

A study by Kauppinen et al. in 2021 tested phages as potential agents for the biocontrol of an opportunistic pathogen *Pseudomonas aeruginosa *in water and showed a significant development of bacteriophage preparations to control pathogens and harmful microbes in water environments [21,22]. A study has demonstrated the effect of bacteriophages on actinomycetes, these are bacteria present in ordinary soil and other organic matter that can grow on water pipes and give off a musty, earthy smell and taste, suggesting the use of bacteriophages for water purification, disinfecting and removing the odour of water, unlike chlorination [23]. Periasamy and Sundaram did a research on hospital wastewater what showed a 100% removal of pathogens from sewage water within 14 hours of incubation [11].

Bacteriophages and water quality were linked. According to this study, all highly satisfactory samples had bacteriophages, followed by that of satisfactory, and minimum in the not satisfactory class of water. According to this study, the odds of diarrhoea were four times higher in the water sample without bacteriophage than in the water sample in which bacteriophage was present.

Strength and limitations

This study supports the potential role of bacteriophages in improving the quality of water, thereby reducing the incidence of diarrhoea. The limitation of this study is that a community-based cross-sectional design was used, therefore, a cause-and-effect relationship can not be established. Hence a prospective study is needed to establish the causal pathway. The best evidence can be obtained through a randomised controlled trial.

## Conclusions

There is a direct association between the presence of bacteriophages and the quality of drinking water.* *The presence of bacteriophages against E. coli, Klebsiella, and Vibrio indicates that the environment under reference was contaminated with these organisms.Further, the prevalence of diarrhoea was significantly more in under-five children having unsatisfactory quality of drinking water with less bacteria-specific phages. 
